# A Bubble-Free Microfluidic Device for Easy-to-Operate Immobilization, Culturing and Monitoring of Zebrafish Embryos

**DOI:** 10.3390/mi10030168

**Published:** 2019-02-28

**Authors:** Zhen Zhu, Yangye Geng, Zhangyi Yuan, Siqi Ren, Meijing Liu, Zhaozheng Meng, Dejing Pan

**Affiliations:** 1 Key Laboratory of MEMS of Ministry of Education, School of Electronic Science and Engineering, Southeast University, Nanjing 210096, China; gengyangye@seu.edu.cn (Y.G.); zy-yuan16@mails.tsinghua.edu.cn (Z.Y.); ren.siqi.43s@st.kyoto-u.ac.jp (S.R.); 2 Model Animal Research Center, Nanjing University, Nanjing 210061, China; liumj@nicemice.cn; 3 Department of Biosystems Science and Engineering, Bio Engineering Laboratory, ETH Zurich, CH-4058 Basel, Switzerland; zhaozheng.meng@bsse.ethz.ch; 4 Cambridge-Suda Genomic Resource Center and Jiangsu Key Laboratory of Neuropsychiatric Diseases, Soochow University, Suzhou 215213, China

**Keywords:** microfluidics, 3D printing, zebrafish embryo, embryogenesis

## Abstract

The development of miniaturized devices for studying zebrafish embryos has been limited due to complicated fabrication and operation processes. Here, we reported on a microfluidic device that enabled the capture and culture of zebrafish embryos and real-time monitoring of dynamic embryonic development. The device was simply fabricated by bonding two layers of polydimethylsiloxane (PDMS) structures replicated from three-dimensional (3D) printed reusable molds onto a flat glass substrate. Embryos were easily loaded into the device with a pipette, docked in traps by gravity, and then retained in traps with hydrodynamic forces for long-term culturing. A degassing chamber bonded on top was used to remove air bubbles from the embryo-culturing channel and traps so that any embryo movement caused by air bubbles was eliminated during live imaging. Computational fluid dynamics simulations suggested this embryo-trapping and -retention regime to exert low shear stress on the immobilized embryos. Monitoring of the zebrafish embryogenesis over 20 h during the early stages successfully verified the performance of the microfluidic device for culturing the immobilized zebrafish embryos. Therefore, this rapid-prototyping, low-cost and easy-to-operate microfluidic device offers a promising platform for the long-term culturing of immobilized zebrafish embryos under continuous medium perfusion and the high-quality screening of the developmental dynamics.

## 1. Introduction

The zebrafish, *Danio rerio*, has become a prominent vertebrate model for disease modeling and drug discovery [1]. Approximately 82% of disease-related human genes have at least one zebrafish orthologue [2]. Zebrafish embryos feature a small size, an optically-transparent body, rapid development, and cost-efficient husbandry, and thus have been increasingly used as ideal model organisms in research areas such as embryogenesis, developmental biology and chemical genetics [3,4,5]. To date, most routine experiments with zebrafish embryos are still performed by means of cell-culturing protocols in conventional microplates, which have several drawbacks [6]. For instance, the static culturing environment of embryos in sample wells may cause accumulative surface absorption of chemical compounds and cross-contamination of embryo metabolites. The water flow for embryos developed in a natural fluid environment cannot be imitated by the static culture. Embryos moving in wells may impose restrictions on the resolution and quality of live imaging.

In the past decade, microfluidics has developed rapidly due to its unique merits, such as small geometric features in micrometer-scale and high surface-to-volume ratio, ability to handle small volume of fluids (microliter to picoliter) in laminar flow regime, and requiring low reagents with a fast response. In addition, the advantages of microfluidics in portability, automation, high throughput, and the ability to integrate multiple functions on a single chip make it an exceptional platform in a variety of fields such as chemical analysis, cell biology, and medicine. Microfluidics can even enhance the propagation, development and potency of some biological organisms, especially in assisted reproductive technology (ART) [7,8].

Furthermore, with the development of advanced manufacturing technologies such as microfabrication, laser micromachining and three-dimensional (3D) printing, dedicated microfluidic devices for the immobilization, flow-perfusion culture, dosing and time-lapse imaging of zebrafish embryos have emerged in the past decade [9,10,11]. A set of microfluidic devices were presented for zebrafish embryos’ culturing and monitoring based on specific modifications in 3D structure design, glass/silicon etching and bonding processes, and medium perfusion [12,13,14]. Although these devices have controllable perfusion systems and enable the culturing of single zebrafish embryos in each chamber, complicated fabrication and operation processes that require dedicated clean-room equipment and instruments limit availability in most biological laboratories, and any movement of embryos in chambers may affect the imaging quality. 

To resolve the problem of embryo movement during optical screening, Akagi et al. developed a microfluidic array with horizontal traps for the immobilization and perfusion of zebrafish embryos [15]. The design and operation mechanism of this array was similar to that of the worm-encapsulated droplet trap array [16], but required a high flow rate of up to 2 mL/min to drag and dock millimeter-scale embryos in traps. Zhu et al. developed a 3D high-throughput microfluidic platform, which enables the stable immobilization of single embryos by combining continuous medium perfusion at a flow rate of 400 µL/min and aspiration via horizontal tunnels embedded between the traps and suction channel [17]. Moreover, in order to avoid the high-flow-rate perfusion that may exert high shear stress on embryos and potentially affect the embryogenesis and embryonic development, laser micromachining was introduced to fabricate a multilayer 3D array of embryo traps with vertical tunnels embedded beneath to immobilize single zebrafish embryos by combined gravitational sedimentation and low-pressure suction [18,19]. However, the surface roughness of vertical traps processed by laser micromachining limited the image resolution and quality of immobilized embryos. Therefore, an easily fabricated and straightforward-to-handle microfluidic system that enables the gentle immobilization and perfusion, stable culturing, and high-resolution imaging of zebrafish embryos is desirable.

In this work, we presented a proof-of-concept microfluidic device for the immobilization, culturing and imaging of zebrafish embryos. The device was comprised of a flat glass substrate and two layers of polydimethylsiloxane (PDMS) structures replicated from 3D printed masters. A bottom PDMS layer was constructed with an embryo-culturing channel to load embryos and five traps to capture embryos for long-term culturing and real-time imaging during their development. Taking advantage of the gas permeability of PDMS materials, a degassing chamber patterned in the top PDMS layer was used to remove air bubbles from the fluidic channel and traps in the bottom PDMS layer by applying a vacuum in the degassing chamber. Computational fluid dynamics (CFD) simulations were performed to estimate the shear stress on the immobilized embryos that were perfused under the continuous medium in the device. Culturing and monitoring of zebrafish embryonic development was carried out over 20 h to verify the performance of the microfluidic device.

## 2. Materials and Methods 

### 2.1. Microfluidic Device

**Figure 1:**
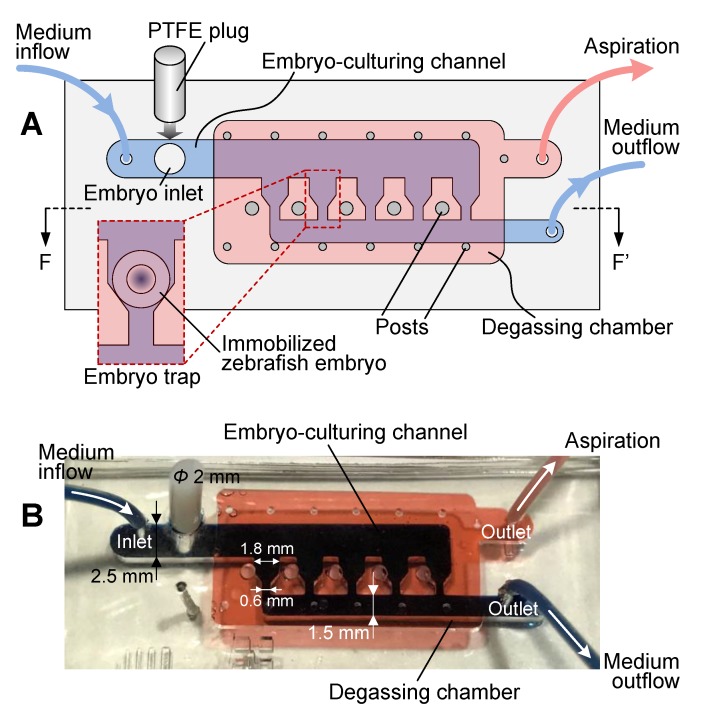
Overview of the microfluidic device for zebrafish embryo immobilization, culturing and monitoring. (**A**) Schematic of the microfluidic device with peripheral interfaces for embryo loading and trapping, culturing-medium perfusion, and degassing. (**B**) Device photo illustrating the embryo-culturing channel (dark blue), the degassing chamber (red), and the polytetrafluoroethylene (PTFE) plug (white) inserted in the embryo inlet.

The microfluidic device consisted of a glass substrate and two PDMS layers patterned with microstructures (Figure 1). The bottom layer, i.e., the embryo-culturing channel, was designed to trap and culture zebrafish embryos. It was composed of an inlet for embryo loading, an inlet and an outlet for medium perfusion, and five horizontal-funnel-like traps for embryo immobilization. Considering that the diameter of zebrafish embryos with chorion is about 1.2 mm, the embryo inlet was 2 mm in diameter, the embryo-culturing channel was 2.5 mm wide and 2 mm high, and the trap featured a wide opening of 1.8 mm and a narrow opening of 0.6 mm to dock and retain an embryo without being flushed away. After the loading of embryos in the device, the embryo inlet was inserted with a 2 mm diameter polytetrafluoroethylene (PTFE) plug to avoid liquid leakage during culturing-medium perfusion. The top PDMS layer, which was irreversibly bonded onto the upper surface of the bottom PDMS layer, was a degassing chamber with several posts to support it. The degassing chamber had a height of 1 mm and fully covered the embryo-culturing channel and traps. 

**Figure 2:**
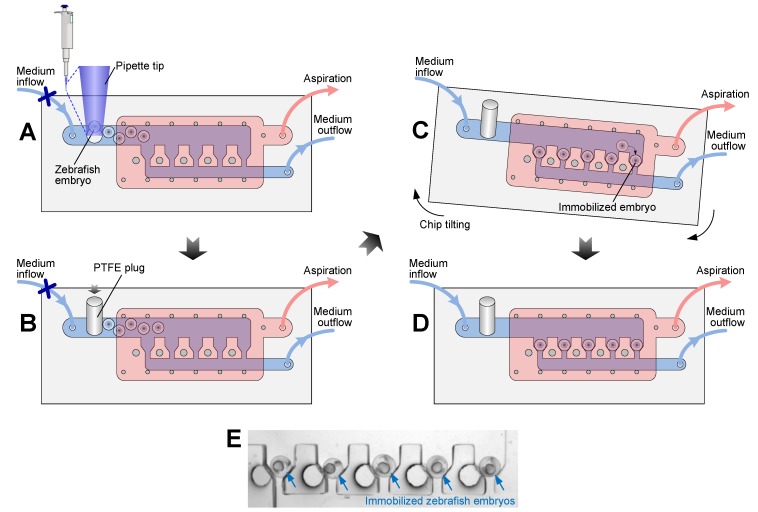
Operation process of the microfluidic device for embryo loading, trapping, and medium perfusion. (**A**) Loading embryos through the embryo inlet using a pipette. (**B**) Blocking the embryo inlet with the PTFE plug. (**C**) Tilting the device to roll embryos in the embryo-culturing channel and dock one embryo in each trap. (**D**) Retaining embryos in traps for culturing and real-time monitoring. (**E**) A micrograph showing five embryos immobilized in the device.

The operation process of the microfluidic device is schematically shown in Figure 2. To load embryos into the device, a 1 mL pipette tip was cut to form a big opening (>1.2 mm) so that embryos could be sucked up and released easily without being damaged. Then, embryos could be transferred into the embryo-culturing channel through the embryo inlet using a pipette (Figure 2A). Afterwards, a 2 mm diameter PTFE plug was inserted into the embryo inlet so that no liquid leakage occurred during medium perfusion (Figure 2B). The medium inflow was then started by controlling a syringe pump. To immobilize embryos, the device was tilted slightly to roll the embryos in the channel and gravitationally dock them in the traps (Figure 2C). The space of each trap could house only one embryo to ensure single-embryo immobilization. After docking, embryos were perfused continuously with the culturing medium to stably maintain the immobilization status by hydrodynamic forces (Figure 2D,E). During the whole process of embryo loading, immobilization and culturing, a vacuum was always applied to the degassing chamber via aspiration to remove any air bubbles from the fluidic channel. 

### 2.2. Device Fabrication

**Figure 3:**
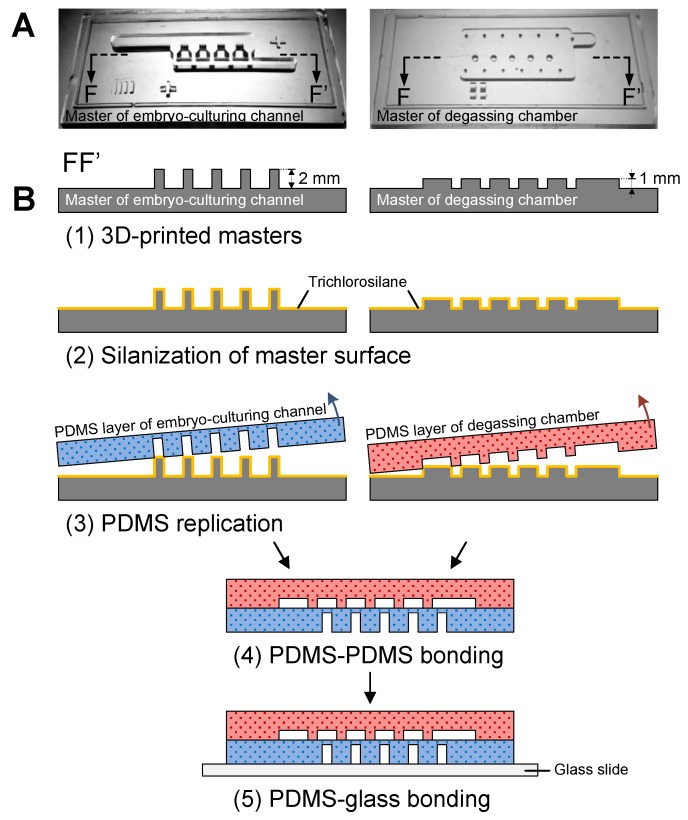
Fabrication process of the microfluidic device. (**A**) Photos of 3D printed masters for embryo-culturing channel and degassing chamber. (**B**-1) Schematic side view along FF’ of Figure 1A and Figure 3A. (**B**-2) Surface silanization of masters with the trichlorosilane. (**B**-3) PDMS replication from masters. (**B**-4) Bonding the degassing chamber onto the embryo-culturing channel. (**B**-5) Bonding the PDMS stamp onto the glass slide.

**Figure 4:**
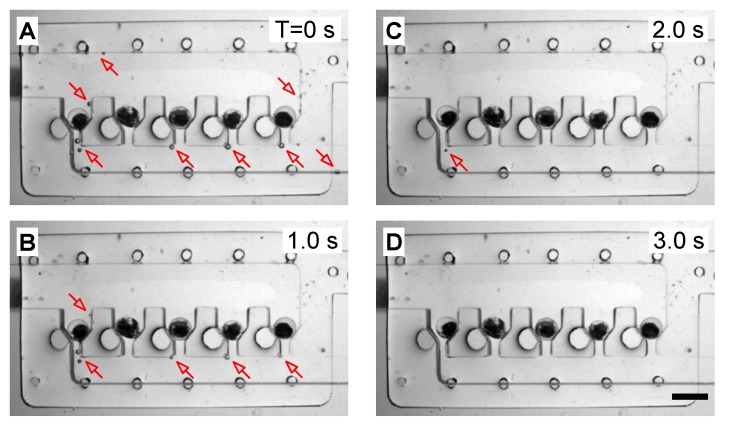
Fast evacuation of air bubbles (indicated by arrows) in the embryo-culturing channel within 3 s. (**A**) T = 0 s; (**B**) T = 1.0 s; (**C**) T = 2.0 s; (**D**) T = 3.0 s. Scale bar: 2 mm.

**Figure 5:**
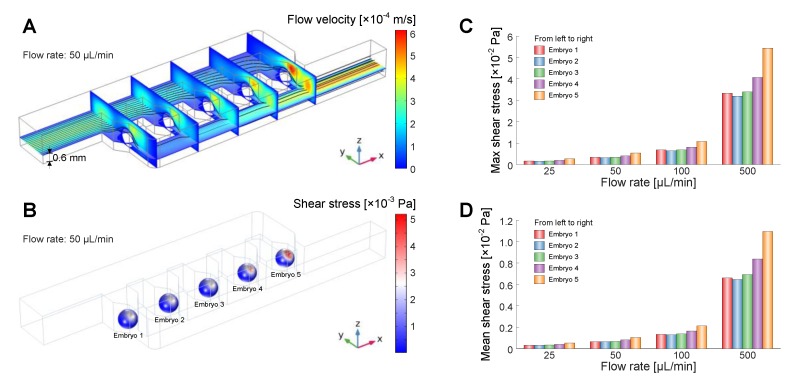
Computational fluid dynamics (CFD) simulations of flow velocity and shear stress for embryo immobilization in the microfluidic device. (**A**) Contour plots and stream lines of cross-sections in the *xy*-plane (across the horizontal middle of immobilized embryos, i.e., 0.6 mm above the channel bottom) and *yz*-plane (across the vertical middle of each immobilized embryo) showing the flow velocity across the embryo-culturing channel immobilized with five embryos. Flow rate was set to 50 µL/min in simulation. (**B**) Contour plot of shear stress over the surfaces of five immobilized embryos. Flow rate was set to 50 µL/min in simulation. (**C**,**D**) Simulated maximum and mean values of shear stress over each embryo at applied flow rates: 25, 50, 100 and 500 μL/min.

The microfluidic device was simply fabricated using a glass-PDMS multi-layer process. Since zebrafish embryos have diameters of around 1.2 mm and require a millimeter- and submillimeter-scale feature of microstructures for entrapment, the standard SU-8-based soft-lithography process was not feasible. Herein, we used an affordable 3D printer (MiiCraft+, MiiCraft, Hsinchu, Taiwan) with its proprietary photopolymer resin (BV007, MiiCraft) to fabricate molds for PDMS replication [20,21,22,23]. Masters for both the embryo-culturing channel and degassing chamber were designed using SolidWorks software (Dassault Systèmes, Waltham, MA, USA). The printer features an *x*-*y* resolution of about 56 μm and a z resolution of 50 μm, which was defined by the minimum upward step of the stage. For each layer of resin with a thickness of 50 μm, the ultraviolet (UV) exposure time was set to 5 s. After printing, the master was soaked in ethanol for 5 min then rinsed with fresh ethanol for another 1 min, followed by UV post-curing for 20 min. After post-curing, the master was soaked in ethanol again for 2 h then baked on a hotplate at 60 °C for 12 h to finalize the master fabrication (Figure 3A,B-1). Both masters were then silanized with trichloro(1H,1H,2H,2H-perfluorooctyl)silane (Sigma-Aldrich, Saint Louis, MO, USA) in vapor phase to prevent PDMS (Sylgard 184, Dow Corning, Midland, MI, USA) from adhering to resin molds (Figure 3B-2). Afterwards, masters were transferred to PDMS with a mixture of 10:1 w/w base to curing agent (Figure 3B-3). After peeling the PDMS replicas from masters, the degassing chamber was first punched with a hole as the inlet for aspiration and subsequently bonded onto the upper surface of the PDMS embryo-culturing channel (Figure 3B-4) through oxygen plasma surface modification (PDC-002-HP, Harrick Plasma, Ithaca, NY, USA). Then, the bonded PDMS stamp was punched with holes as inlets and outlets for embryo loading and medium perfusion. Lastly, the PDMS stamp was irreversibly bonded to a bare glass slide (Figure 3B-5) to finalize the device fabrication.

### 2.3. Computational Fluid Dynamics (CFD) Modelling and Simulations

In order to investigate the fluid dynamics for trapping and maintaining embryos and shear stress exerted on the immobilized embryos in the microfluidic device, 3D CFD simulations were performed using COMSOL Multiphysics software (COMSOL Inc., Burlington, MA, USA) using ‘Laminar Flow’ physics from the CFD Module. Geometric structures and parameters in the model were derived from the microfluidic device in Figure 1. According to the geometric parameters and applied flow rate of 50 μL/min, the Reynolds number in the embryo-culturing channel was calculated to be 0.37, which confirmed that the microfluidic device followed the laminar flow regime. Embryos immobilized by the traps were simply modelled as rigid and non-deformable spheres in the fluidic domain. Subdomains were assigned with a density of 1000 kg/m^3^ and a dynamic viscosity of 0.001 Pa∙s (for water at 20 °C). For the incompressible fluid under laminar flow regime that follows the Navier–Stokes equation, the pressure and flow velocity were conducted by the governing equations as follows:

(1) $$\rho \left(u\cdot \nabla \right)u=\nabla \cdot [-pI+\mu \left(\nabla u+{\left(\nabla u\right)}^{T})\right]+F$$

(2) ρ∇·(u)=0

where ρ is density, **u** is velocity vector, p is pressure, **I** is unit matrix, μ is dynamic viscosity, and **F** is volume force vector. 

A no-slip boundary condition was applied to the channel walls and the sphere surfaces. Laminar inflow with different volumetric flow rates of 25, 50, 100 and 500 μL/min was applied to the inlet boundary of the fluidic channel, and a pressure of 0 Pa was set to the outlet boundary. Due to the limitation of physical memory and time–cost in the numerical calculation, a predefined meshing process with normal element size was applied to all domains. Supplementary Table S1 lists the detailed parameters of element size set for meshing, such as maximum and minimum element size, and maximum element growth rate. Supplementary Figure S1 illustrates the translucent profiles of the microfluidic structures without and with spheres after meshing. The shear stress (*τ*) over the sphere surfaces was calculated by using the shear stress components in *x*-, *y*- and *z*-directions on the basis of the following equation:

(3) $$\tau =\sqrt{\tau_{x}^{2}+\tau_{y}^{2}+\tau_{z}^{2}}$$

After simulations, contours and stream lines referring to the flow velocity in the fluidic channel and contours of the shear stress over the sphere surfaces were obtained. 

### 2.4. Zebrafish Embryo Trapping, Culturing and Imaging

Embryos were obtained from natural spawning of wild type adult zebrafish and were collected in E3 medium (5 mM NaCl, 0.17 mM KCl, 0.33 mM CaCl_2_, and 0.33 mM MgSO_4_). After flushing the microfluidic channel with E3 medium, we used a pipette with a modified tip to transfer embryos into the embryo-culturing channel through the embryo inlet, which was then plugged up with a PTFE pillar. The microfluidic device was manually tilted in order to dock embryos in the traps, then fixed on the stage of a stereomicroscope (NSZ-608T, Novel Optics, Nanjing, China) with adhesive tape. For embryo culturing, E3 medium, initially loaded in a 100 mL syringe affixed on a syringe pump, was perfused into the embryo-culturing channel via PTFE tubing at a constant flow rate of 50 µL/min, and the room temperature was kept at 28.5 °C. In order to remove any bubbles from the embryo-culturing channel, the degassing chamber was connected to the house vacuum supplied by a vacuum pump (MPC 1201 T, Ilmvac, Germany), which can provide an ultimate pressure of below 2 mbar according to its datasheet. Time-lapse imaging of embryonic development was performed using the stereomicroscope equipped with a digital camera (E3CMOS, ToupTek Photonics, Hangzhou, China) and running its camera control software (ToupView, ToupTek Photonics) for image acquisition at a time interval of 150 s. 

## 3. Results and Discussion

### 3.1. Degassing

During the long-term culturing and monitoring of zebrafish embryos, any air bubbles brought to the embryo-culturing channel and traps may squeeze the immobilized embryo out of its original position or obstruct the view of imaging, and thus affect the stability of embryo immobilization and the quality of time-lapse imaging. As such, we took advantage of the gas permeability of PDMS materials and removed air bubbles from the fluidic channel by applying a vacuum to the degassing chamber. Herein, we tested the degassing chamber, which was assigned to remove air bubbles attached to PDMS channel walls in the embryo-culturing channel. Once a low pressure (vacuum) was exerted over the degassing chamber via aspiration, a high-pressure difference was generated between the air in bubbles and the degassing chamber. Thus, air in the bubbles could be aspirated to pass through the gas-permeable PDMS and ultimately removed. Figure 4 shows the experimental results. Initially, there were some air bubbles attached to the surface of the embryo-culturing channel, as indicated by arrows in Figure 4A. After switching on the vacuum supply, bubbles started to reduce in size and number (Figure 4B,C) and finally disappeared within 3 s (Figure 4D). The result demonstrated that the degassing chamber was capable of removing air bubbles highly efficiently, thereby enabling the stable culturing and monitoring of zebrafish embryonic development.

### 3.2. CFD Simulations

3D CFD simulations were performed to study the fluidic dynamics for embryo immobilization and the shear stress exerted on the immobilized embryos in the microfluidic device. Figure 5A shows the flow velocity across the embryo-culturing channel immobilized with five embryos. The stream lines indicate that the liquid flowed over each immobilized embryo and became trapped towards the outlet. The contour plots of vertical cross-sections indicate that the maximum value of flow velocity existed in the rightmost trap. As a result, the shear stress over the surface of the rightmost embryo exhibited a maximum value of 5.2 × 10^−3^ Pa among the five immobilized embryos perfused at 50 µL/min (Figure 5B). Based on the CFD simulations, we then obtained the maximum and mean shear stress over each embryo at different flow rates: 25, 50, 100 and 500 μL/min (Figure 5C,D). With the increase of flow rate, both maximum and mean values of shear stress showed an increase trend. When applying a flow rate of 100 μL/min, the maximum shear stress of the rightmost embryo exceeded 0.01 Pa. At a flow rate of 500 μL/min, the mean shear stress of the same embryo also exceeded 0.01 Pa. The CFD simulations thus indicated that a high flow rate resulted in high shear stress over the surfaces of embryos. Previous studies [15,18] have verified that the maximum shear stress in the order of 0.01 Pa has negligible influence on developing embryos due to the protection of the robust chorion membrane. In the experiment, we found that immobilized embryos during culturing were occasionally displaced from their original positions when a flow rate of 30 µL/min was applied for medium perfusion. This phenomenon can be elucidated by the fact that a flow rate lower than 30 µL/min cannot provide sufficient hydrodynamic forces to retain the embryos stably in their traps. Considering the required stable embryo immobilization and low shear stress over the embryos during long-term culturing and imaging, we, therefore, set the flow rate of medium perfusion to 50 µL/min in the experiment. 

### 3.3. Zebrafish Embryonic Development

We then evaluated the microfluidic device as a stable platform to culture the immobilized zebrafish embryos and monitor their dynamic development. The immobilized embryos were perfused with E3 medium at a constant flow rate of 50 µL/min for a long-term culture (over 20 h). Meanwhile the embryonic development was traced by time-lapse imaging at a time interval of 150 s. 

**Figure 6:**
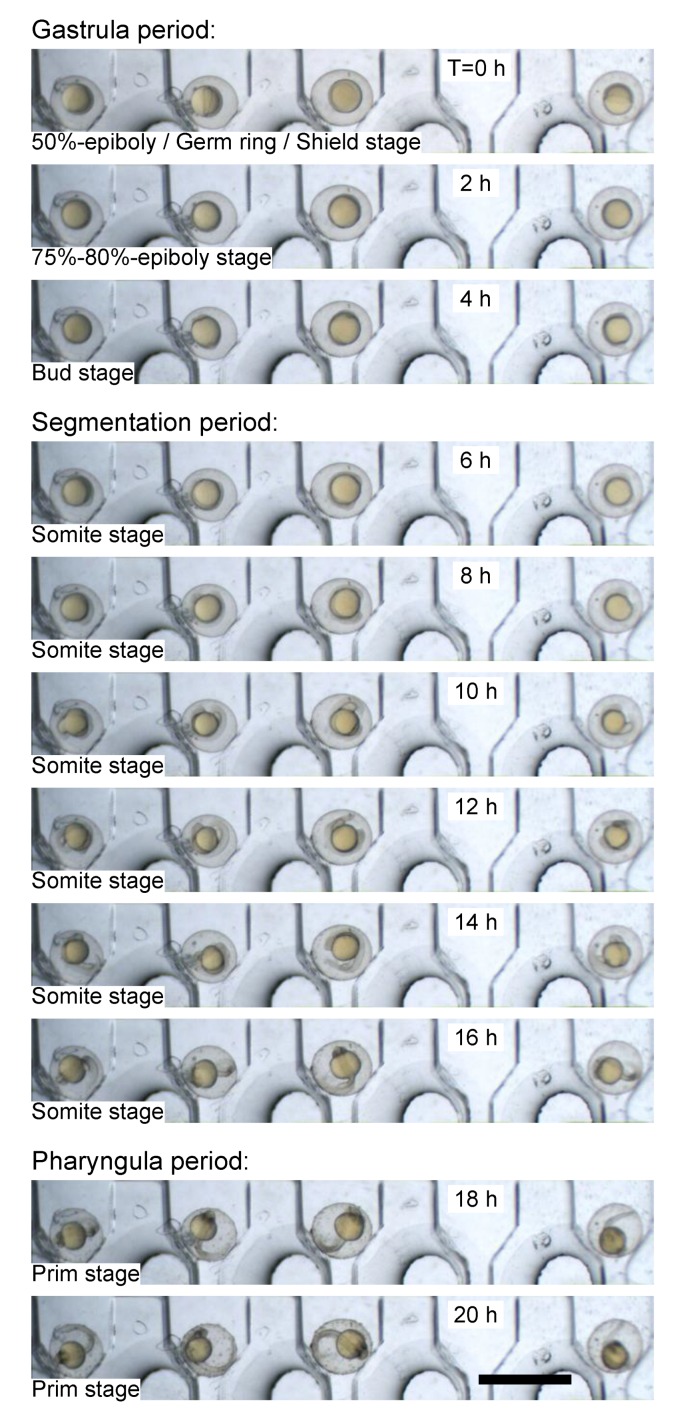
Twenty-hour live imaging of the early-stage development of zebrafish embryos immobilized in the microfluidic device. Scale bar: 2 mm.

Figure 6 shows photographs every 2 h of the four recorded zebrafish embryos, which developed healthily and uniformly during the recording time course. Image acquisition started around 5 h post fertilization (T = 0 h) and the embryonic development had already entered the gastrula stage. The epiboly displaced the blastoderm margin and remained at 50%. The germ ring and the embryonic shield could be observed. Then, the epiboly continued until the yolk plug was completely covered by the blastoderm, and a tail bud became visible (4 h, bud stage). From this stage onward, the development of embryos entered the segmentation period. During this development period (6–16 h), somites appeared sequentially and developed in the trunk and tail, which elongated and became more prominent, and the early rudiments of primary organs and body movement could be observed. Afterwards, the embryos entered the pharyngula period for further morphogenetic development (18 h). Therefore, the aforementioned experimental results confirmed the capabilities of the microfluidic device for stable immobilization and long-term culturing of zebrafish embryos and monitoring their early-stage development without interfering in the intricate embryogenesis.

## 4. Conclusions

We have presented a rapid-prototyping, low-cost and easy-to-operate microfluidic device for stable immobilization, long-term culturing and high-quality imaging of zebrafish embryos. The device has been simply fabricated by bonding a glass substrate with two layers of PDMS replicas from the 3D printed reusable masters. Experimental results have demonstrated that zebrafish embryos can be easily loaded into the embryo-culturing channel with a pipette and then rolled into the traps in turn under gravity by manually tilting the device. Suggested by the CFD simulations, we have optimized the embryo-trapping regime and diminished the potential shear stress exerted over the immobilized embryos. Continuous medium perfusion at a low flow rate of 50 µL/min provided adequate hydrodynamic forces to ensure the stable immobilization of the embryos. Air bubbles in the embryo-culturing channel and traps were rapidly and effectively removed by the degassing chamber on top. As a result, the zebrafish embryos were stably immobilized and underwent long-term time-lapse monitoring of their dynamic development. Culturing and monitoring over 20 h during the early stages of the embryonic development of the zebrafish successfully confirmed the functions of the microfluidic device. Therefore, the microfluidic platform promises to perform the stable immobilization, continuous medium perfusion, and long-term culturing of more zebrafish embryos with a potential scaling-up of the device, allowing for high-quality monitoring of dynamic embryonic development. 

## Supplementary Materials

The following are available online at https://www.mdpi.com/2072-666X/10/3/168/s1, Figure S1: Translucent meshing profiles of the microfluidic structures (A) without and (B) with spheres. Table S1: Element size parameters in meshing of 3D CFD simulation.
