# Supplementary material for: A Bubble-Free Microfluidic Device for Easy-to-Operate Immobilization, Culturing and Monitoring of Zebrafish Embryos
Source: Micromachines (Basel). 2019 Feb 28;10(3):168. doi: 10.3390/mi10030168 (PMC6470713; doi:10.3390/mi10030168)
Supplement: Supplementary file 1 [file micromachines-10-00168-s001.pdf]

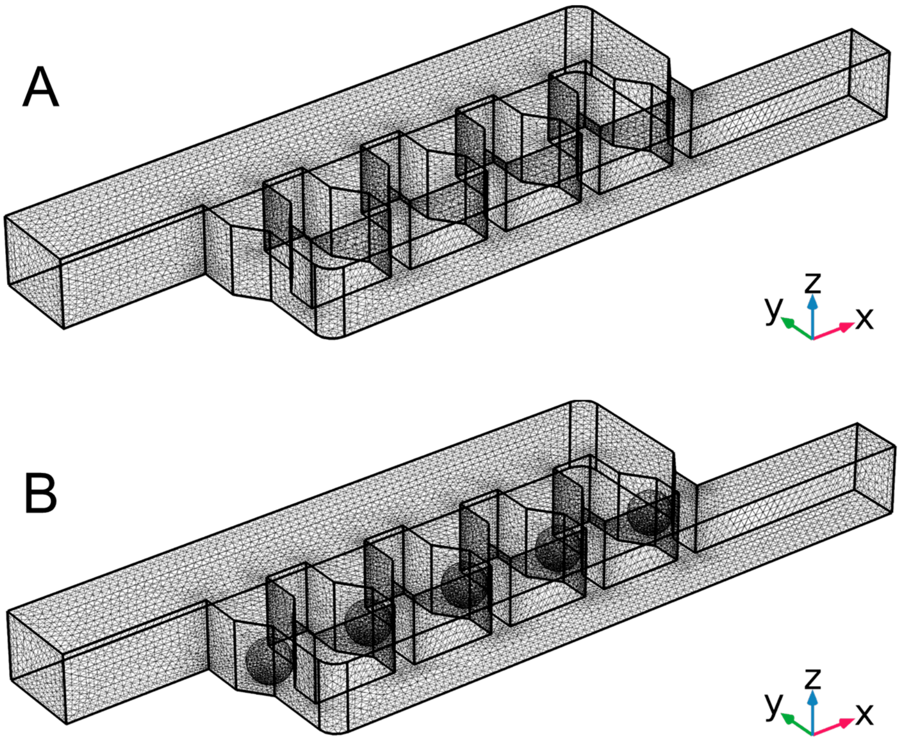

Figure S1. Translucent meshing profiles of the microfluidic structures (**A**) without and (**B**) with spheres.

Table S1. Element size parameters in meshing of 3D CFD simulation.

Element Size Parameters	Setting Values
Maximum element size	2.49×10^{-4}
Minimum element size	7.43×10^{-5}
Maximum element growth rate	1.15
Curvature factor	0.6
Resolution of narrow regions	0.7
